# Network analysis of gut microbial communities reveal key genera for a multiple sclerosis cohort with Mycobacterium avium subspecies paratuberculosis infection

**DOI:** 10.1186/s13099-024-00627-7

**Published:** 2024-07-10

**Authors:** Hajra Ashraf, Plamena Dikarlo, Aurora Masia, Ignazio R. Zarbo, Paolo Solla, Umer Zeeshan Ijaz, Leonardo A. Sechi

**Affiliations:** 1 Department of Biomedical Sciences, University of Sassari, Sassari, Italy; 2 BIOMES NGS GmbH, Schwartzkopffstraße 1, 15745, Halle 21, Wildau, Germany; 3 Department of Medicine and Pharmacy, Neurology, University of Sassari, Sassari, Italy; 4 Water & Environment Research Group, Mazumdar-Shaw Advanced Research Centre, University of Glasgow, Glasgow, UK; 5 National University of Ireland, Galway, University Road, Galway, Ireland; 6 Department of Molecular and Clinical Cancer Medicine, University of Liverpool, Liverpool, UK; 7 Complex Structure of Microbiology and Virology, AOU Sassari, Sassari, Italy

**Keywords:** Multiple sclerosis, Gut microbiome, Network inference, Integrated value of influence

## Abstract

**Background:**

In gut ecosystems, there is a complex interplay of biotic and abiotic interactions that decide the overall fitness of an individual. Divulging the microbe-microbe and microbe-host interactions may lead to better strategies in disease management, as microbes rarely act in isolation. Network inference for microbial communities is often a challenging task limited by both analytical assumptions as well as experimental approaches. Even after the network topologies are obtained, identification of important nodes within the context of underlying disease aetiology remains a convoluted task. We therefore present a network perspective on complex interactions in gut microbial profiles of individuals who have multiple sclerosis with and without *Mycobacterium avium subspecies paratuberculosis* (MAP) infection. Our exposé is guided by recent advancements in network-wide statistical measures that identify the keystone nodes. We have utilised several centrality measures, including a recently published metric, Integrated View of Influence (IVI), that is robust against biases.

**Results:**

The ecological networks were generated on microbial abundance data (*n* = 69 samples) utilising 16 S rRNA amplification. Using SPIEC-EASI, a sparse inverse covariance estimation approach, we have obtained networks separately for MAP positive (+), MAP negative (-) and healthy controls (as a baseline). Using IVI metric, we identified top 20 keystone nodes and regressed them against covariates of interest using a generalised linear latent variable model. Our analyses suggest *Eisenbergiella* to be of pivotal importance in MS irrespective of MAP infection. For MAP + cohort, *Pyarmidobacter*, and *Peptoclostridium* were predominately the most influential genera, also hinting at an infection model similar to those observed in Inflammatory Bowel Diseases (IBDs). In MAP- cohort, on the other hand, *Coprostanoligenes group* was the most influential genera that reduces cholesterol and supports the intestinal barrier.

**Conclusions:**

The identification of keystone nodes, their co-occurrences, and associations with the exposome (meta data) advances our understanding of biological interactions through which MAP infection shapes the microbiome in MS individuals, suggesting the link to the inflammatory process of IBDs. The associations presented in this study may lead to development of improved diagnostics and effective vaccines for the management of the disease.

**Supplementary Information:**

The online version contains supplementary material available at 10.1186/s13099-024-00627-7.

## Background

Multiple sclerosis (MS) is a chronic inflammatory and neurodegenerative disorder [1] that affects brain and spinal cord impacting around 2.5 million people worldwide [2]. The origin of demyelination and inflammation is not clear yet however interplay between environmental and genetic factors are known to develop MS [3, 4]. Growing literature on this topic highlights the role of gut microbiota as a strong environmental influencer in the MS context [5]. It is proposed that perturbations in the gut microbiota could stimulate proinflammatory responses that serve as additional mechanism in the pathogenesis of MS [6, 7]. On the other hand, several studies have linked Mycobacterium avium subspecies paratuberculosis (MAP) infections with MS [8, 9]. MAP is a versatile intracellular parasite that colonizes intraepithelial macrophages in the mucosa-associated lymphoid tissue of the small intestine. It can induce chronic granulomatous gastroenteritis, known as John’s disease or paratuberculosis, in animals, especially ruminants [10]. Various molecular and serological tests have reported the presence of MAP in the blood of individuals with multifactorial diseases, including type 1 diabetes (T1D) [11, 12], Crohn’s disease (CD) [13], multiple sclerosis (MS) [8, 9] and Parkinson’s disease (PD) [14]. Molecular mimicry is known to be one of the potential mechanisms by which MAP triggers autoimmune diseases due to the structural similarity of MAP antigens to self-antigens [15]. Despite the extensive research conducted on the gut microbiota and MS there is hardly any study that identified microbes or their functions linked to MS specially when infected by MAP.

In a complex microbial ecosystem, species rarely act alone. Either they strive for resources following a competitive exclusion principle with one species outcompeting another, or they live in symbiosis, or there are predator-prey interactions. Recovery of both biotic and abiotic interactions then leads to understanding how stable the ecosystem is [16], with the network of interactions able to reveal the important species functioning in the ecosystem. Typically, in microbial networks, through in situ analytical approaches, highly interacting nodes called hubs are identified [17]. In a previous study, identified hubs or keystone nodes were then later confirmed experimentally [18], and were deemed to be important. Furthermore, these inferential network-based approaches have found use in clinical applications. For example, in [19], highly connected hub species associated better with the clinical changes (as compared to highly abundant and prevalent species) in cystic fibrosis patients with chronic lung infections.

In general, once a network topology is obtained, network-wide statistical measures such as centralities are calculated which, ascertain how central a node is within the expanse of a network. For example, *Degree Centrality*, *Cluster Rank*, *Betweenness*, *Collective Influence*, *Network Neighborhood*, and *Local H-index* are some centrality measures that can quantify the importance of nodes. Whilst each one of these on their own can serve to highlight a particular nuance, a more sophisticated approach that can simultaneously consider a set of centrality measures covering local and global features of network can offer better synergy by reducing biases inherent with some of the measures. For this purpose, a more sophisticated network-wide statistical measure called Integrated Value of Influence (IVI) [20] is proposed that uses six important centrality measures (as above) as building blocks to derive *Hubness* and *Spreading* scores eventually combining them to a single IVI measure. This single measure then enables recovery of the most important topological characteristics of the network identifying keystone nodes that have biological relevance with the measure robust against biases. The aim of this paper is to then to consolidate these recent advancements in network statistics to identify keystone nodes in multiple sclerosis patients with and without MAP infection. We then associate these keystone nodes with the anthropometric and sociodemographic information. For association, we utilize the *Generalized Linear Latent Variable Model* (GLLVM) [21] approach where the abundance of individual microbes is regressed against covariates of interest by also incorporating a small number of latent variables. The fitted beta coefficients through the GLLVM approach then gives directionality (positive or negative association) against the covariates of interest consolidating the clinical or environmental context under which the data is generated. However, fitting GLLVM against sources of variability when there are thousands of taxa, significantly more than the number of samples, is computationally challenging and impractical for larger datasets. IVI leverages this by ranking taxa in terms of their influence, thus allowing exploration of the top most influential nodes, enabling better convergence of the likelihood in the reduced space of the variables.

## Methods

### Bioinformatics

Our previous study [22] provides a comprehensive overview of the study design, stool sampling, their processing, and bioinformatic analysis. In brief, this study involved comparative analysis of three distinct study groups; MS patients who tested positive for MAP infection (MAP+), MS patients who tested negative for MAP infection (MAP-) and a control group consisting of healthy individuals. Each participant provided two samples, labelled as T1 and T2 typically collected a month apart. A total of 97 individuals were screened for participation in this study at the Multiple Sclerosis Center of the University of Cagliari, Italy. The collected samples underwent 16 S rRNA amplicon sequencing using V3-V4 primer set on an Illumina MiSeq instrument. Out of the 97 stool samples collected from the participants, only 74 had provided a sufficient DNA yield for microbiome analyses. An additional 5 samples were excluded due to low read numbers (< 5000 reads), resulting in a total of 69 samples included in final analyses.

### Network inference

We have used an OTU table of *n* = 69 x *P* = 16,787 OTUs (see [22]) where VSEARCH pipeline [23] was used to construct OTUs at 99% similarity threshold. The summary statistics of reads mapping to these OTUs for samples as follows: [Minimum: 5,074; 1st Quartile: 14,380; Median: 18,060; Mean: 20,634; 3rd Quartile: 22,651; Maximum: 96,572]. After obtaining the taxonomy of OTUs using SILVA SSU Ref NR database release v.138 [24], the abundances of OTUs belonging to the same genus were collated together giving an *n* = 69 x *P* = 128 dimensional genera table. Note that where the OTUs were not resolved at genus level, they were put in the “__Unknown__” category. From the 69 samples, we inferred the network separately for Healthy Control (*n* = 24), MAP+ (*n* = 27), and MAP- (*n* = 18) cohorts. We have used the SPIEC-EASI [25] approach using the standard parameters in the function spiec.easi(abundance_table, method=’mb’, lambda.min.ratio = 1e-2, nlambda = 20, pulsar.params = list(rep.num = 50)), where abundance_table is the table extracted separately for Healthy Control (HC), MAP + and MAP- individuals.

### Network wide statistics

Having obtained the network topology for all three cohorts (Healthy Control, MAP+, and MAP- MS patients), we have calculated several network wide statistics using the R packages igraph [26], influential [20], and centiserve [27]. We have used the statistics given in Supplementary Table S1, with comparative analyses of these statistics for different cohort given in Supplementary Figure S1 (Supplementary_Materials.docx).

### Generalised Linear Latent Variable Model (GLLVM)

To find the relationship between most influential nodes [top 20 selected based on Integrated View of Influence (IVI) metric] and the sources of variation (*Sex*, *Age*, *BMI*, *Time Points*, *Weight Change*, *Having Children*, *Having Pets*, *Smoker*, *Work Routine*, *Sports*, *Leisure Time*, *Sleep Duration*, *Antibiotics*, *Sweet Consumption*, *Drinking Water*, *Alcohol Consumption*, *Probiotics Consumption*, *Stool Consistency*, and *Disease Duration* [only available for MAP+, and MAP- cohort]), we have used Generalised Linear Latent Variable Model (GLLVM) [21] which extends the basic generalized linear model that regresses the mean abundances ${\mu }_{ij}$ (for $i$-th sample and $j$-th microbe) of individual microbes against environmental covariates ${x}_{i}$ as above by incorporating latent variables (confounders) ${u}_{i}$ as $g\left({\mu }_{ij}\right)={\eta }_{ij}={\alpha }_{i}+{\beta }_{0j}+{\varvec{x}}_{i}^{T}{\varvec{\beta }}_{j}+{\varvec{u}}_{i}^{T}{\varvec{\theta }}_{j}$, where ${\varvec{\beta }}_{j}$ are the microbe specific coefficients associated with individual covariate. A 95% confidence interval of ${\varvec{\beta }}_{j}$ whether positive (increasing the abundance of microbe) or negative (decreasing the abundance of microbe), and not crossing 0 boundary gives directionality with respect to a particular covariate. ${\varvec{\theta }}_{j}$ are the corresponding coefficients associated with latent variable. ${\beta }_{0j}$ are microbes’ specific intercepts, whilst ${\alpha }_{i}$ are optional sample effects which can either be chosen as fixed effects or random effects (not used in this study). To model the distribution of individual microbes, we have used Negative Binomial distribution. Additionally, the approximation to the log-likelihood is done through Variational Approximation (VA) with final sets of parameters in glvmm() function being family = ‘negative.binomial’, method="VA”, control.start=list(n.init = 7, jitter.var = 0.1) that converged the optimization algorithm associated with GLLVM for HC, MAP+ and MAP- cohort.

## Results

### Recovered keystone nodes vary between HC, MAP + and MAP- cohort

**Fig. 1:**
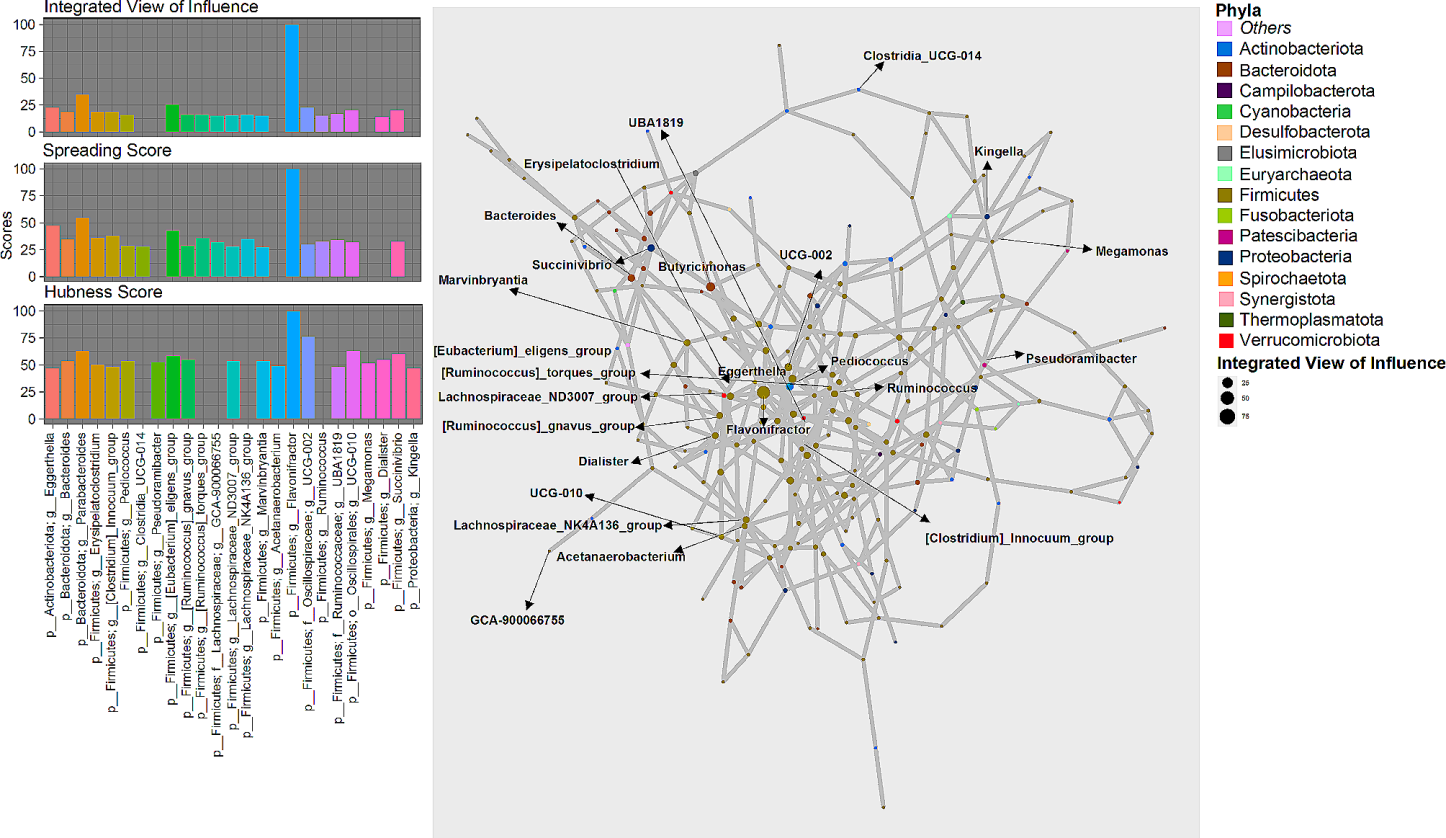
Network inferred for HC samples using SPIEC-EASY algorithm for OTUs collated at genus level. The size of the nodes in the network corresponds to *Integrated View of Influence* scores whilst the nodes are colored at Phylum level. Left panel shows the top 20 important nodes along with their scores based on composite measure *Integrated View of Influence*, along with the *Spreading Score* and the *Hubness Score*. To improve clarity, bars of nodes that were not in top 20 list for a given metric were not drawn. These influential genera (*n* = 25) are then annotated on the network figure shown in the middle panel

**Fig. 2:**
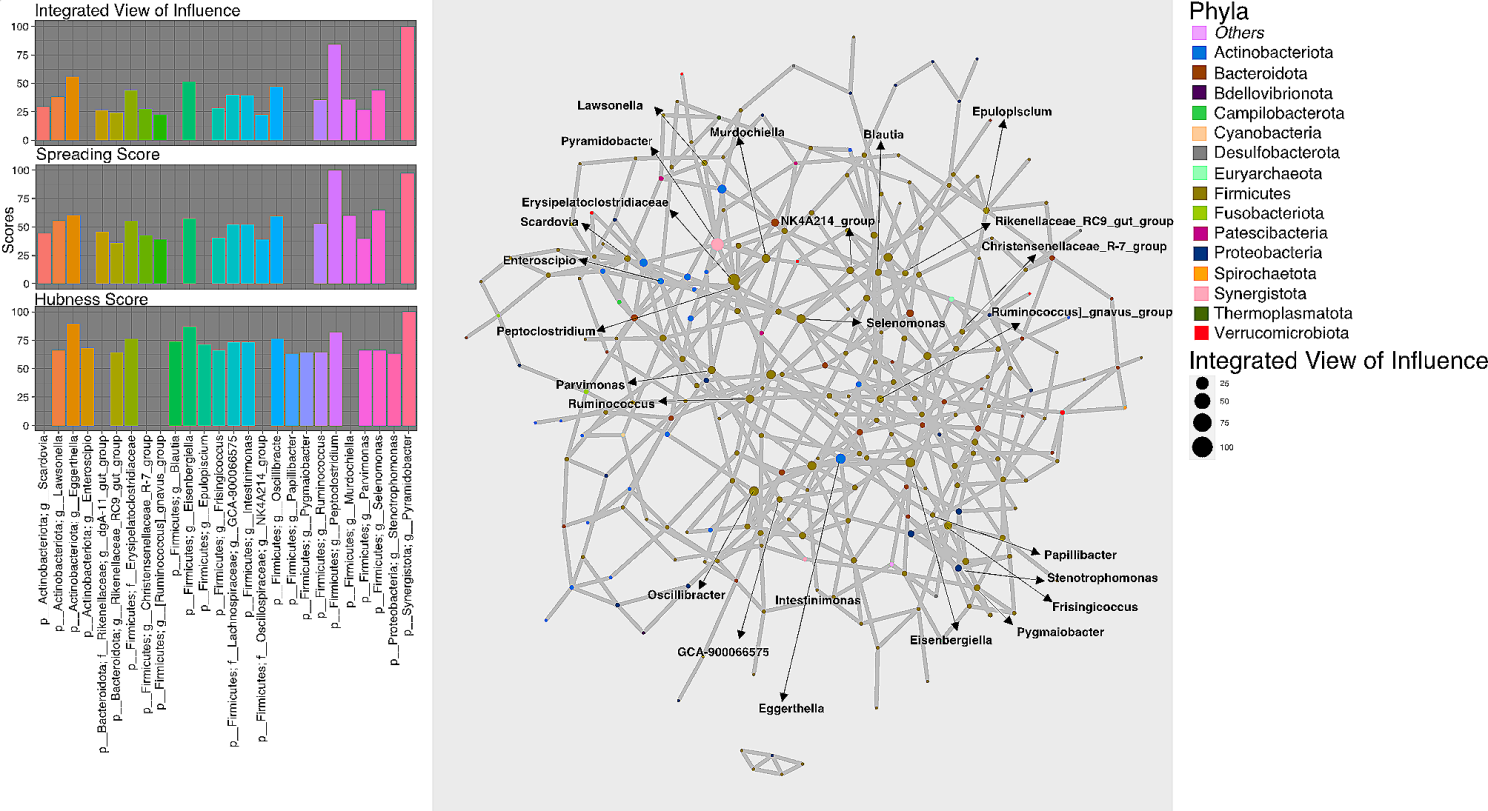
Network inferred for MAP + samples using SPIEC-EASY algorithm for OTUs collated at genus level. The size of the nodes in the network corresponds to *Integrated View of Influence* scores whilst the nodes are colored at Phylum level. Left panel shows the top 20 important nodes along with their scores based on composite measure *Integrated View of Influence*, along with the *Spreading Score* and the *Hubness Score*. To improve clarity, bars of nodes that were not in top 20 list for a given metric were not drawn. These influential genera (*n* = 26) are then annotated on the network figure shown in the middle panel

**Fig. 3:**
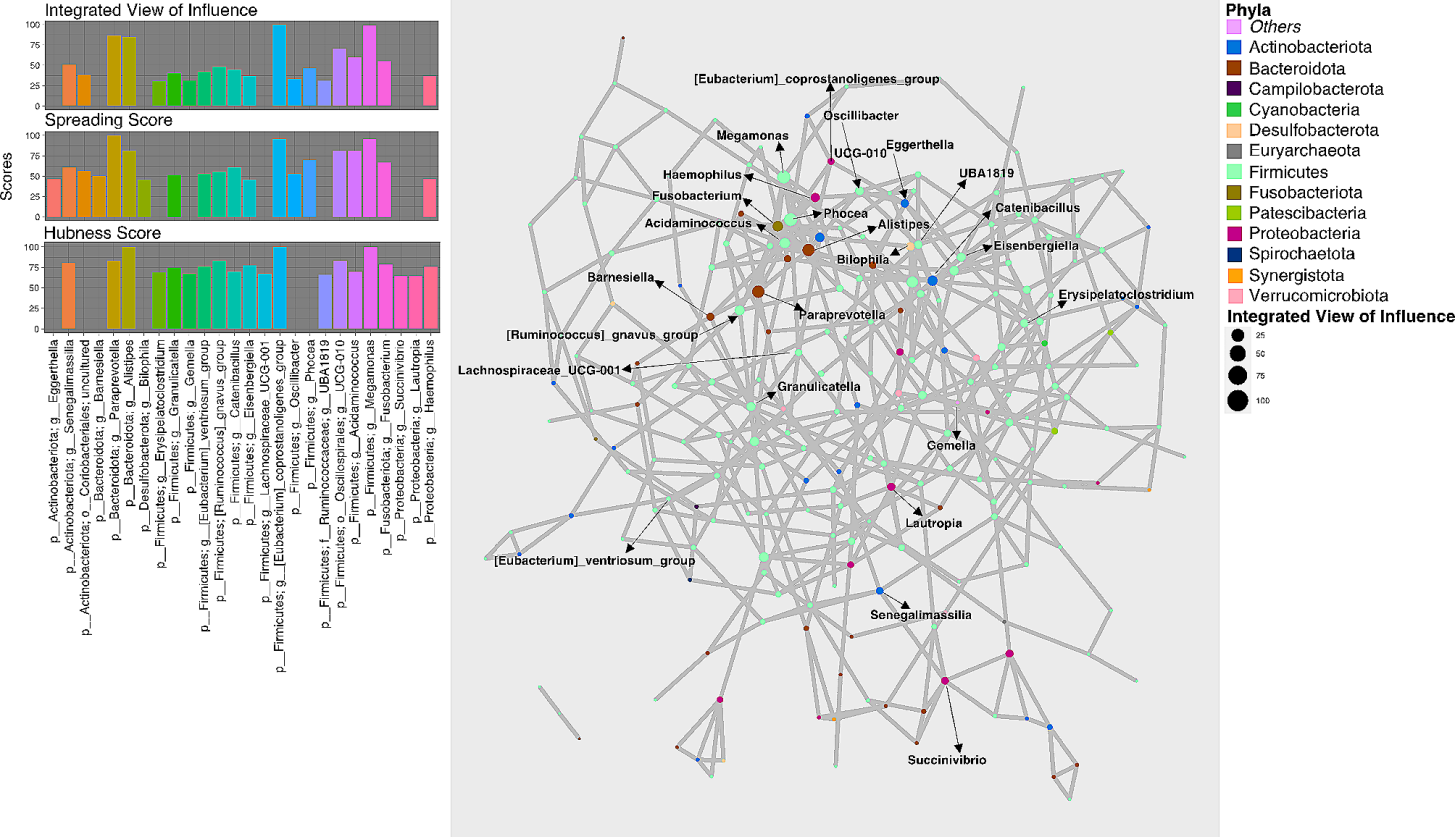
Network inferred for MAP- samples using SPIEC-EASY algorithm for OTUs collated at genus level. The size of the nodes in the network corresponds to *Integrated View of Influence* scores whilst the nodes are colored at Phylum level. Left panel shows the top 20 important nodes along with their scores based on composite measure *Integrated View of Influence*, along with the *Spreading Score* and the *Hubness Score*. To improve clarity, bars of nodes that were not in top 20 list for a given metric were not drawn. These 26 influential genera (after combining the results from all metrics) are then annotated on the network figure shown in the middle panel

The networks of genera inferred for HC, MAP + and MAP- cohorts are shown in Figs. 1, 2 and 3 along with the statistics for top influential nodes. Although we have used several network wide metrics, we mainly emphasized on three strategies for the identification of key stone genera: (a) **Spreading Score**: which itself a combination of four metrics i.e., Neighbourhood connectivity (NC), Cluster rank (CR), Betweenness centrality (BC) and Collective influence (CI) that are used to identify the potential genera having higher spreading potential within the microbiome network. (b) **Hubness Score**: It is used to identify genera that have high centrality measures [(Degree centrality (DC_i_) and Local H-index (LH_index_)] and (c) **Integrated View of Influence (IVI)**: It integrates both spreading and hubness score as a single metric and identifies microbial network most influential nodes.

We have taken a union of the top 20 nodes selected for either of the three metrics, Spreading Score, Hubness Score, and IVI. These are shown in the left panels of Figs. 1, 2 and 3 and selected 25, 26, and 26 genera for HC, MAP+, and MAP- cohorts, respectively. Majority of the influential nodes belonged to the phylum *Firmicutes* (21 for HC, 18 for MAP+, and 15 for MAP-) with the most influential nodes according to IVI being: *Flavonifractor* (HC); *Pyramidobacter* (MAP+); and *[Eubacterium]_coprostanoligenes_group* (MAP-). Of all the influential nodes, *Eggerthella* and *[Ruminococcus]_gnavus_group* were selected for all three cohorts. Amongst other common nodes: *GCA_900066575 (Lachnospiraceae)* and *Ruminococcus* are common between HC and MAP+; *Erysipelatoclostridium*, *UCG_010 (Oscillospirales)*, *Megamonas*, and *Succinovibrio* are common between HC and MAP-; and *Eisenbergiella and Oscillibacter* are common between MAP + and MAP-.

Supplementary Data Table S1 contains all interactions recovered for each cohort where, genera (top 5 for each cohort) that achieved very high IVI scores are highlighted along with their interacting secondary connections. These come out to be 14 unique genera with *Eggerthella* common between MAP + and HC cohort. Secondary connections of the top 5 IVI nodes that are common between MAP + and MAP- are *Aldercreutzia*, *[Ruminococcus]_gnavus_group, Oscillobacter*, and *Acidaminococcus*. Secondary connections of the top 5 IVI nodes had a high degree of overlap between HC and MAP+, and these include *Flavonifractor*, *[Clostridium]_innocuum_group*, *Eisenbergiella*, *Colidextribacter*, and *Ruminococcus*.

### Top 20 influential keystone nodes based on IVI and their relationship with the clinical parameters and the exposome

**Fig. 4:**
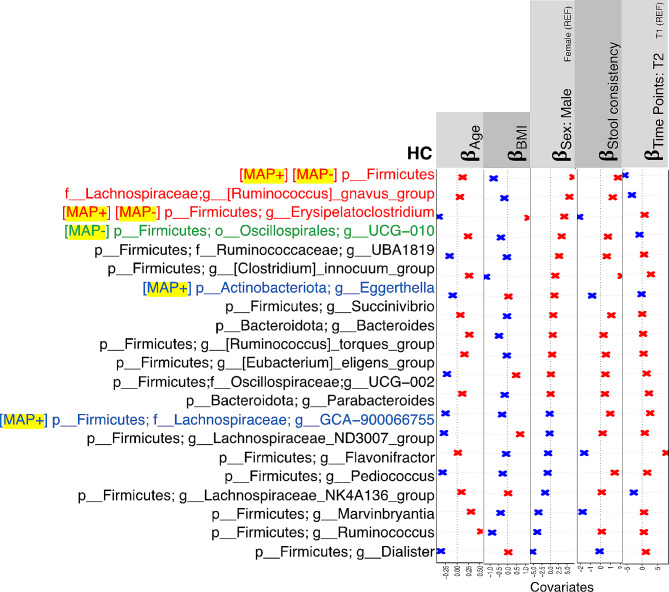
𝜷− coefficients returned from GLLVM procedure for intrinsic covariates considered in this study, and the top 20 influential nodes returned for HC samples in Fig. 1 using IVI metric, with the complete results including extrinsic parameters shown in Supplementary Figure S2. Those coefficients which are positively associated with the microbial abundance of a particular genera are represented in red color whilst those that are negatively associated are represented with blue color, respectively. Non-significant associations, if any, are represented with the black color. For categorical variables, one level acts as a reference and is annotated with REF. Genera also found influential for MAP + and MAP- cohort are represented with red color, whilst those found in either of MAP- and MAP + cohort are represented with green and blue colors, respectively

**Fig. 5:**
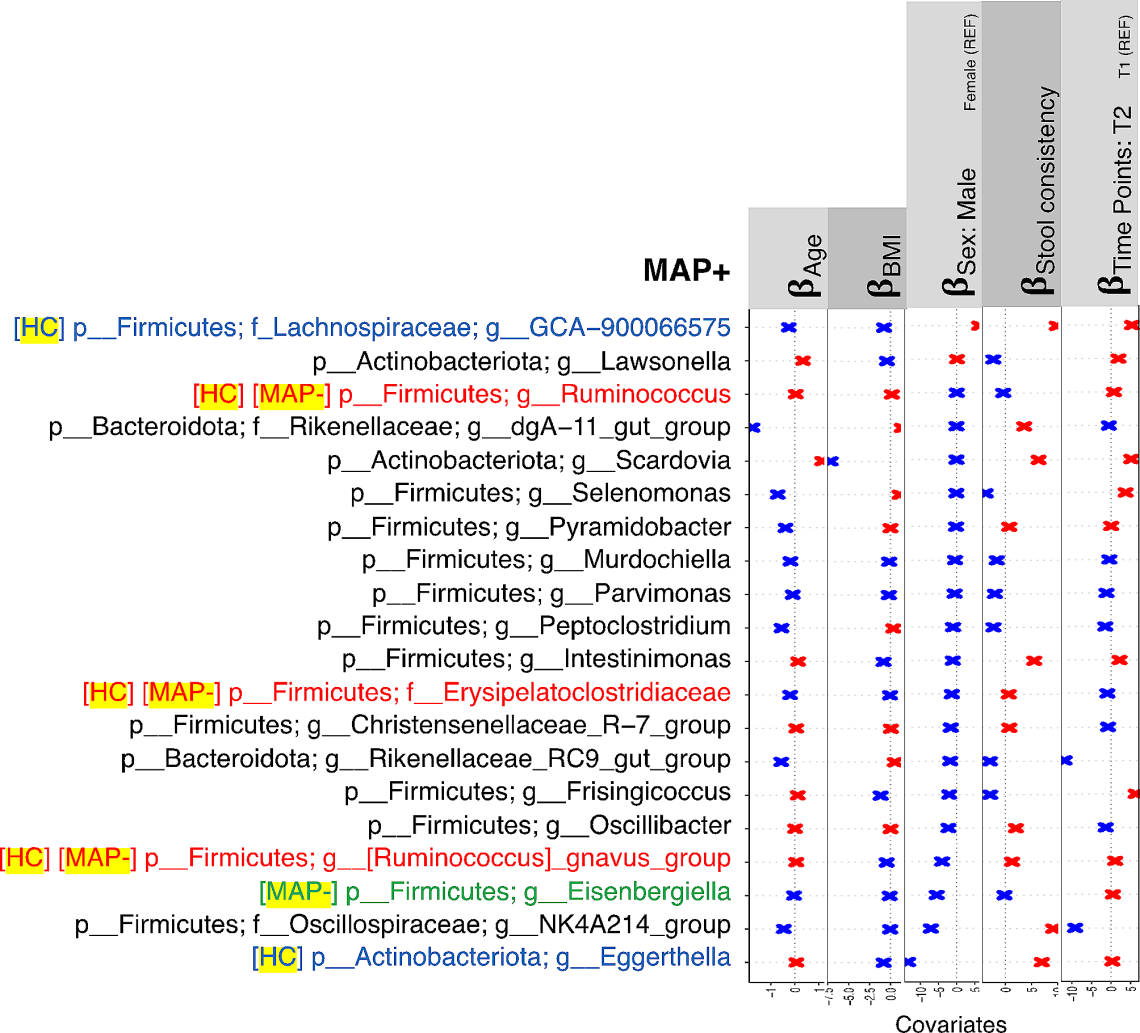
𝜷− coefficients returned from GLLVM procedure for intrinsic covariates considered in this study, and for the top 20 most influential nodes returned for MAP + samples in Fig. 2 using IVI metric, with the complete results including extrinsic parameters shown in Supplementary Figure S3. Those coefficients which are positively associated with the microbial abundance of a particular genera are represented in red color whilst those that are negatively associated are represented with blue color, respectively. Non-significant associations, if any, are represented with the black color. For categorical variables, one level acts as a reference and is annotated with REF. Genera also found influential for HC and MAP- cohort are represented with red color, whilst those found in either of MAP- and HC cohort are represented with green and blue colors, respectively

**Fig. 6:**
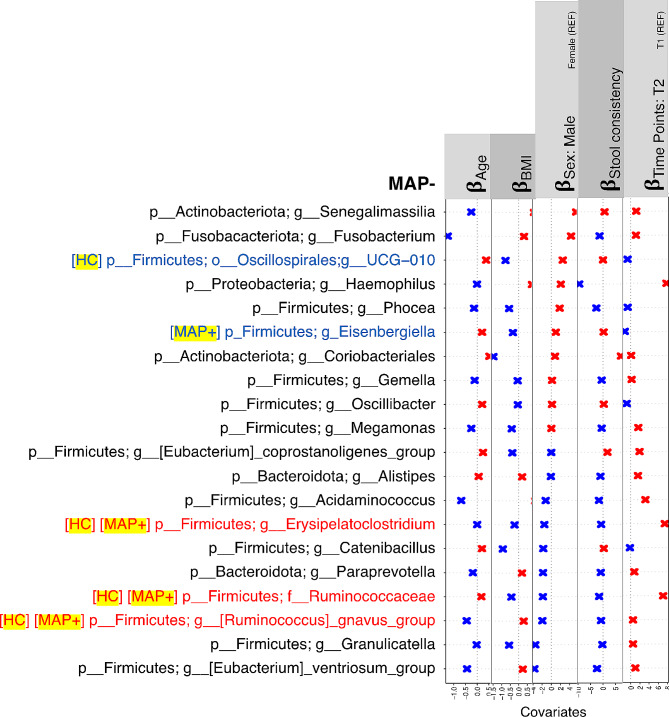
𝜷− coefficients returned from GLLVM procedure for intrinsic covariates considered in this study, and for the top 20 most influential nodes returned for MAP- samples in Fig. 3 using IVI metric, with the complete results including extrinsic parameters shown in Supplementary Figure S4. Those coefficients which are positively associated with the microbial abundance of a particular genera are represented in red color whilst those that are negatively associated are represented with blue color, respectively. Non-significant associations, if any, are represented with the black color. For categorical variables, one level acts as a reference and is is annotated with REF. Genera also found influential for HC and MAP + cohort are represented with red color, whilst those found in either of HC and MAP + cohort are represented with blue color

We then employed a GLLVM to regress the top 20 most influential genera against different sources of variation. These associations are shown in Figs. 4 and 5, and 6. The covariates include *Sex*, *Age*, *BMI*, *Time Points*, *Weight Change*, *Having Children*, *Having Pets*, *Smoker*, *Work Routine*, *Sports*, *Leisure Time*, *Sleep Duration*, *Antibiotics*, *Sweet Consumption*, *Drinking Water*, *Alcohol Consumption*, *Probiotics Consumption*, *Stool Consistency*, and *Disease Duration*, with the information provided by the subjects at the time of sample collection.

There are some keystone nodes that are common across all cohorts. These include *Ruminococcus* genera in general, and also include *[Ruminococcus]_gnavus_group* and *Erysipelatoclostridium*. Also, *Eisenbergiella* is the only genera which is influential in MS cohort, irrespective of MAP infection. For *Eisenbergiella*, using GLLVM, some of the covariates differ in their association between MAP + and MAP- cohort. These include *Age*, *Probiotics consumptions (> 1 year,3–6 months)*, *Sex(male)*, *Sleep duration*, *Smoker*, *Stool consistency*, *Weight change (Loss)*, and *Work routine (sitting)* which are all positively associated with *Eisenbergiella* in MAP- individuals whilst they are negatively associated with *Eisenbergiella* in MAP + individuals. Also, *drinking water > 2 L*, *Leisure time(normal)*, *probiotics consumption(currently)*, *sweet consumption 5–6 per week*, and *work routine (sedentary, standing)* are negatively associated with *Eisenbergiella* for MAP- individuals and positively associated with *Eisenbergiella* for MAP + individuals.

To identify the important genera in the networks associated with MAP+, MAP- and HCs, we have employed network-based statistics based on centrality, connectivity and hubness scores/strategies. Supplementary Figure S1 shows comparison of network-wide statistics among all the study cohorts. The statistics revealed that MAP- individuals have the highest IVI scores than MAP + and HCs. The trend is same in terms of *Hubness score*, *Local H-index*, *Laplacian centrality*, *Spreading score* and *Geodesic K-path centrality*. However, for the MAP + group, *Neighborhood connectivity*, *Lin centrality* and *Latora closeness* is highest as compared to the other cohorts. These measures suggest that in MAP + networks, microbial genera are closer, central and have more connections to neighborhood genera as compared to the MAP- and HC cohorts.

## Discussion

The human microbiome exhibits significant diversity in its composition, interconnections, and resilience both within and across individuals. Based on the interconnectedness of microbes, our aim is to identify keystone microbes associated with diifferent clinical parameters. The “keystone” concept has its roots in microbial ecology that designate a species with a significant role on community relative to its abundance. The concept has been extended to microbial abundance data where certain microbial species may play a crucial role in shaping the community structure and function. Although these species are few and far between, they have a markedly increased influence. Rahman and Schomberg et al., [28] elaborates on this further and utilized this approach in identifying important enzymes in microbial networks. Furthermore, their results highlighted high “between centrality” values relative to node degree as a means to identify “choke points” that play a significant role in carrying out fundamental metabolic conversions in bacteria and act as central hubs in metabolic networks influencing various interaction and pathways.

Using IVI, a composite approach built from several centrality measures, we identified the top 3 most influential nodes for MAP + cohort which are *Pyramidobacter*, *Peptoclostridium*, and *Eggerthella*. *Eggerthella*, a microbe associated with cysteine degradation [29] has been associated with multiple sclerosis although its causal role is not fully established yet [30]. The genus *Pyramidobacter* comprises strains that are anaerobic, non-motile, asaccharolytic bacilli producing acetic and isovaleric acids along with small quantities of propionic and isobutyric acids [31]. It is thought to enhance fiber degradation and may depend on thiamine to grow [32]. Though the direct link of *Pyramidobacter* with MS remains unclear, it was previously associated with 17-Hamilton Depression Rating Scale (HAMD-17) assessment [33].

The genera that are typically affected by thiamine supplementation including those that were found important for MAP + cohort include *Erysipelotrichaceae*, *Lachnospiraceae*, *Selenomonas, Pyramidobacter, Christensenellaceae R7* and, *Ruminococcaceae NK4A214*. *Pyramidobacter* species are vital cellulolytic bacteria and produce acetate as the main fermentation product [34], the enhanced *Pyramidobacter* by thiamine could support the fiber degradation. Our findings of acetate producers implicated in MAP + cohort is in line with literature [35] where based on metabolomics, higher concentration of acetate is observed for MAP infection predominantly in males. It is worth noting that *Peptoclostridium*, a causative agent of diarrhea and colitis [36] adds another dimension to the complex interplay of microbial species in the gut. Its potential implications in MS individuals with MAP infection further adds credence to the association of Multiple Sclerosis (MS) with Inflammatory Bowel Disease (IBD) [37, 38] particularly Crohn’s disease and Ulcerative colitis. Additionally, genome wide association studies have revealed a shared risk locus between IBD and MS indicating a common underlying pathological mechanism affecting both conditions [39]. Therefore, one would expect the microbial signature between MS and IBD to be similar. In individuals without MAP infection, there is abundance of *Eubacterium coprostanoligenes, Megamonas Ruminococcus gnavus* and, *Alistipes*. *Eubacterium coprostanoligenes* is known for its capability of transforming cholesterol to coprostanol [40]. It plays a major role in stimulating lactic acid metabolism toward the production of SCFAs, that support the intestinal barrier [41]. This process also leads to secondary bile acids secretions which play a role in the balance between health and disease particularly in association with inflammatory bowel disease [42]. *Megamonas* found to be increased in MS [43], which is also implicated in Amyotrophic lateral sclerosis (ALS) [44]. *Alistipes* are differentially abundant in RRMS (Relapse Remitting Multiple Sclerosis) [45] that signifies its potential in immune related responses. *Ruminococcus gnavus* is known to produce a polysaccharide that induces TNFα production emphasizing its role in immune modulation [46].

In our study, we have also identified interaction within each cohort focusing on those genera with very high IVI scores. In MAP + cohort, a noticeable cooccurrence relationship occurs between the genera *Murdochiella* and *Pyramidobacter*. This observation is in line with a study conducted by Caudet et al. [47], which confirmed the higher relative abundance of these two species in a similar context. Similarly, *Eubacterium eligens* and *Intestinimonas* are identified as cooccurring species. Both are regarded as butyriciproducens that produce SCFA, especially butyric acid [48]. Interestingly, these species were stimulated in fermentations from patients with IBD [49]. *Faecalibacterium* and *Intestinimonas* has been considered as potential probiotics for treating and alleviating inflammatory bowel disease [50], although *Intestinimonas* could potentially increase in abundance in animal models of inflammation [51]. Nonetheless, it’s important to consider potential complexities, as indicated by a study that found an inverse relationship between *Rikenellaceae* and *Pyramidobacter* [52]. In MAP- individuals, *Bifidobacterium* and *Alistipes* were identified as cooccurring *species*, with this behavior also observed in individuals with *Parkinson’s Disease* [53]. The overlap of our observed co-occurrence patterns with other neurological conditions gives credence to network-based approaches to understanding disease etiology.

Furthermore, we have also implemented GLLVM regression model to investigate the association of the keystone nodes with the covariates of interest. Notably, *Eisenbergiella*, a gram negative, non-motile, non-spore producing bacteria demonstrated distinct association with individuals characterized as MAP + and MAP- considering various covariates of interest. These covariates include *Age*, *Probiotics consumptions (> 1 year,3–6 months)*, *Sex(male)*, *Sleep duration*, *Smoker*, *Stool consistency*, *Weight change(Loss)*, and *Work routine (sitting)*, *Drinking water > 2 L*, *Leisure time(normal)*, *probiotics consumption(currently)*, *sweet consumption 5–6 per week*, and *work routine (sedentary, standing).* Our findings suggests that modulation of *Eisebergiella* abundance as a biocontrol agent can potentially be useful in clinical settings.

In conclusion, our research explores the complex microbial landscape for microbe-microbe interactions within individuals affected by multiple sclerosis (MS), with a specific focus on those with Mycobacterium avium subspecies paratuberculosis (MAP) infection. Incorporating genera identified through the IVI statistic led to interesting associations for both MAP + and MAP- groups. Particularly, *Pyramidobacter*, *Peptoclostridium*, and *Eggerthella* in the MAP + cohort, and their potential metabolic nuances may pave the way for development of therapeutic agents. The downstream GLLVM regression model further elucidated the association of *Eisenbergiella* with various covariates of interests, suggesting a potential link between its abundance and factors i.e., age, probiotic consumption, and lifestyle. However, our conclusions are drawn based on a limited sample size. Further research work including a larger cohort, with temporal sampling will unravel further associations between microbes that may have been missed in this study, and will lead to development of microbial modulation strategies that might have beneficial effects.

### Electronic supplementary material

Below is the link to the electronic supplementary material.

**Supplementary Material 1.** Details on the network-wide statistics used including supplementary figures

**Supplementary Material 2.** Co-occurrence network obtained at genera level for HC, MAP+, and MAP− cohorts. Furthermore, top 5 interacting genera are highlighted for each cohort

**Supplementary Material 3.** Network-wide statistics for HC network

**Supplementary Material 4.** Network-wide statistics for MAP+ network

**Supplementary Material 5.** Network-wide statistics for MAP− network

## Data Availability

Sequence data that support the findings of this study have been deposited in the European Nucleotide Archive with the primary accession code PRJEB67783.

## Abbreviations

16SrRNA: 16 S ribosomal RNA
MS: Multiple sclerosis
PD: Parkinson’s disease
MAP: Mycobacterium avium subspecies paratuberculosis
OTUs: Operational Taxonomic Units
T1D: Type 1 diabetes
CD: Crohn’s disease
IVI: Integrated Value of Influence
GLLVM: Generalised Linear Latent Variable Model
NC: Neighbourhood connectivity
CR: Cluster rank
BC: Between centrality
DC_i_: Degree centrality
LH_index_: Local H-index
CI: Collective influence

## References

[1] Thompson AJ, Baranzini SE, Geurts J, Hemmer B, Ciccarelli O (2018). Multiple sclerosis. Lancet.

[2] Tullman MJ (2013). Overview of the epidemiology, diagnosis, and disease progression associated with multiple sclerosis. Am J Manag Care.

[3] Goodin DS, Khankhanian P, Gourraud P-A, Vince N (2021). The nature of genetic and environmental susceptibility to multiple sclerosis. PLoS ONE.

[4] Marrie RA (2004). Environmental risk factors in multiple sclerosis aetiology. Lancet Neurol.

[5] Chen J, Chia N, Kalari KR, Yao JZ, Novotna M, Paz Soldan MM (2016). Multiple sclerosis patients have a distinct gut microbiota compared to healthy controls. Sci Rep.

[6] Durack J, Lynch SV (2019). The gut microbiome: relationships with disease and opportunities for therapy. J Exp Med.

[7] Kadowaki A, Quintana FJ (2020). The gut–CNS axis in multiple sclerosis. Trends Neurosci.

[8] Ekundayo TC, Olasehinde TA, Falade AO, Adewoyin MA, Iwu CD, Igere BE, Ijabadeniyi OA (2022). Systematic review and meta-analysis of Mycobacterium avium subsp. paratuberculosis as environmental trigger of multiple sclerosis. Multiple Scler Relat Disorders.

[9] Hayashi F, Isobe N, Cossu D, Yokoyama K, Sakoda A, Matsushita T (2021). Elevated mycobacterium avium subsp. paratuberculosis (MAP) antibody titer in Japanese multiple sclerosis. J Neuroimmunol.

[10] Eslami M, Shafiei M, Ghasemian A, Valizadeh S, Al-Marzoqi AH, Shokouhi Mostafavi SK (2019). Mycobacterium avium paratuberculosis and Mycobacterium avium complex and related subspecies as causative agents of zoonotic and occupational diseases. J Cell Physiol.

[11] Sechi LA, Paccagnini D, Salza S, Pacifico A, Ahmed N, Zanetti S (2008). Mycobacterium avium subspecies paratuberculosis bacteremia in type 1 diabetes mellitus: an infectious trigger?. Clin Infect Dis.

[12] Manca Bitti ML, Masala S, Capasso F, Rapini N, Piccinini S, Angelini F et al. Mycobacterium avium subsp. paratuberculosis in an Italian cohort of type 1 diabetes pediatric patients. Journal of Immunology Research. 2012;2012. 10.1155/2012/785262 PMC3400352 22844325

[13] Naser SA, Ghobrial G, Romero C, Valentine JF (2004). Culture of Mycobacterium avium subspecies paratuberculosis from the blood of patients with Crohn’s disease. Lancet.

[14] Arru G, Caggiu E, Paulus K, Sechi GP, Mameli G, Sechi LA (2016). Is there a role for Mycobacterium avium subspecies paratuberculosis in Parkinson’s disease?. J Neuroimmunol.

[15] Cossu D, Cocco E, Paccagnini D, Masala S, Ahmed N, Frau J (2011). Association of Mycobacterium avium subsp. paratuberculosis with multiple sclerosis in sardinian patients. PLoS ONE.

[16] Allesina S, Tang S (2012). Stability criteria for complex ecosystems. Nature.

[17] Paine RT (1966). Food web complexity and species diversity. Am Nat.

[18] Carlström CI, Field CM, Bortfeld-Miller M, Müller B, Sunagawa S, Vorholt JA (2019). Synthetic microbiota reveal priority effects and keystone strains in the Arabidopsis phyllosphere. Nat Ecol Evol.

[19] Layeghifard M, Li H, Wang PW, Donaldson SL, Coburn B, Clark ST (2019). Microbiome networks and change-point analysis reveal key community changes associated with cystic fibrosis pulmonary exacerbations. Npj Biofilms Microbiomes.

[20] Salavaty A, Ramialison M, Currie PD. Integrated value of influence: an integrative method for the identification of the most influential nodes within networks. Patterns. 2020;1(5). 10.1016/j.patter.2020.100052 PMC7660386 33205118

[21] Niku J, Hui FK, Taskinen S, Warton DI (2019). Gllvm: fast analysis of multivariate abundance data with generalized linear latent variable models in r. Methods Ecol Evol.

[22] Ashraf H, Dikarlo P, Masia A, Zarbo I, Solla P, Ijaz U, Sechi L. Mycobacterium avium subspecies paratuberculosis (MAP) infection, and its impact on gut microbiome of individuals with multiple sclerosis. 2023. 10.1038/s41598-024-74975-4 PMC11479286 39402079

[23] Rognes T, Flouri T, Nichols B, Quince C, Mahé F (2016). VSEARCH: a versatile open source tool for metagenomics. PeerJ.

[24] Quast C, Pruesse E, Yilmaz P, Gerken J, Schweer T, Yarza P (2012). The SILVA ribosomal RNA gene database project: improved data processing and web-based tools. Nucleic Acids Res.

[25] Kurtz ZD, Müller CL, Miraldi ER, Littman DR, Blaser MJ, Bonneau RA (2015). Sparse and compositionally robust inference of microbial ecological networks. PLoS Comput Biol.

[26] Csardi G, Nepusz T (2006). The igraph software package for complex network research. InterJournal Complex Syst.

[27] Jalili M, Salehzadeh-Yazdi A, Asgari Y, Arab SS, Yaghmaie M, Ghavamzadeh A, Alimoghaddam K (2015). CentiServer: a comprehensive resource, web-based application and R package for centrality analysis. PLoS ONE.

[28] Rahman SA, Schomburg D (2006). Observing local and global properties of metabolic pathways:‘load points’ and ‘choke points’ in the metabolic networks. Bioinformatics.

[29] Braccia DJ, Jiang X, Pop M, Hall AB (2021). The capacity to produce hydrogen sulfide (H2S) via cysteine degradation is ubiquitous in the human gut microbiome. Front Microbiol.

[30] Cekanaviciute E, Yoo BB, Runia TF, Debelius JW, Singh S, Nelson CA et al. Gut bacteria from multiple sclerosis patients modulate human T cells and exacerbate symptoms in mouse models. Proceedings of the National Academy of Sciences. 2017;114(40):10713-8. 10.1073/pnas.1711235114 PMC5635915 28893978

[31] Downes J, Vartoukian SR, Dewhirst FE, Izard J, Chen T, Yu W-H (2009). Pyramidobacter piscolens gen. nov., sp. nov., a member of the phylum ‘Synergistetes’ isolated from the human oral cavity. Int J Syst Evol MicroBiol.

[32] Pan X, Xue F, Nan X, Tang Z, Wang K, Beckers Y (2017). Illumina sequencing approach to characterize thiamine metabolism related bacteria and the impacts of thiamine supplementation on ruminal microbiota in dairy cows fed high-grain diets. Front Microbiol.

[33] Yao S, Xie H, Wang Y, Shen N, Chen Q, Zhao Y (2023). Predictive microbial feature analysis in patients with depression after acute ischemic stroke. Front Aging Neurosci.

[34] Bainbridge ML, Cersosimo LM, Wright A-DG, Kraft J (2016). Rumen bacterial communities shift across a lactation in Holstein, Jersey and Holstein× Jersey dairy cows and correlate to rumen function, bacterial fatty acid composition and production parameters. FEMS Microbiol Ecol.

[35] Karunasena E, McMahon KW, Chang D, Brashears MM (2014). Host responses to the pathogen Mycobacterium avium subsp. paratuberculosis and beneficial microbes exhibit host sex specificity. Appl Environ Microbiol.

[36] Pereira FL, Oliveira Júnior CA, Silva RO, Dorella FA, Carvalho AF, Almeida GM (2016). Complete genome sequence of Peptoclostridium difficile strain Z31. Gut Pathogens.

[37] Sadovnick A, Paty D, Yannakoulias G (1989). Concurrence of multiple sclerosis and inflammatory bowel disease. N Engl J Med.

[38] Gupta G, Gelfand JM, Lewis JD (2005). Increased risk for demyelinating diseases in patients with inflammatory bowel disease. Gastroenterology.

[39] Yang Y, Musco H, Simpson-Yap S, Zhu Z, Wang Y, Lin X (2021). Investigating the shared genetic architecture between multiple sclerosis and inflammatory bowel diseases. Nat Commun.

[40] Mukherjee A, Lordan C, Ross RP, Cotter PD (2020). Gut microbes from the phylogenetically diverse genus Eubacterium and their various contributions to gut health. Gut Microbes.

[41] Petri RM, Neubauer V, Humer E, Kröger I, Reisinger N, Zebeli Q (2020). Feed additives differentially impact the epimural microbiota and host epithelial gene expression of the bovine rumen fed diets rich in concentrates. Front Microbiol.

[42] Tomova A, Bukovsky I, Rembert E, Yonas W, Alwarith J, Barnard ND, Kahleova H (2019). The effects of vegetarian and vegan diets on gut microbiota. Front Nutr.

[43] Sanchez JMS, DePaula-Silva AB, Libbey JE, Fujinami RS (2022). Role of diet in regulating the gut microbiota and multiple sclerosis. Clin Immunol.

[44] Zeng Q, Shen J, Chen K, Zhou J, Liao Q, Lu K (2020). The alteration of gut microbiome and metabolism in amyotrophic lateral sclerosis patients. Sci Rep.

[45] Reynders T, Devolder L, Valles-Colomer M, Van Remoortel A, Joossens M, De Keyser J (2020). Gut microbiome variation is associated to multiple sclerosis phenotypic subtypes. Ann Clin Transl Neurol.

[46] Henke MT, Kenny DJ, Cassilly CD, Vlamakis H, Xavier RJ, Clardy J. Ruminococcus gnavus, a member of the human gut microbiome associated with Crohn’s disease, produces an inflammatory polysaccharide. Proceedings of the National Academy of Sciences. 2019;116(26):12672-7. 10.1073/pnas.1904099116 PMC6601261 31182571

[47] Caudet J, Trelis M, Cifre S, Soriano JM, Rico H, Merino-Torres JF (2022). Interplay between intestinal bacterial communities and unicellular parasites in a morbidly obese population: a neglected trinomial. Nutrients.

[48] Rosés C, Viadel B, Nieto JA, Soriano-Romaní L, Romo-Hualde A, Agudelo A et al. Gut microbiota modulatory capacity of Brassica oleracea italica x alboglabra (Bimi^®^). 2023.

[49] Calvete-Torre I, Sabater C, Antón MJ, Moreno FJ, Riestra S, Margolles A, Ruiz L (2022). Prebiotic potential of apple pomace and pectins from different apple varieties: modulatory effects on key target commensal microbial populations. Food Hydrocolloids.

[50] Li A, Ding J, Shen T, Han Z, Zhang J, Abadeen ZU (2021). Environmental hexavalent chromium exposure induces gut microbial dysbiosis in chickens. Ecotoxicol Environ Saf.

[51] Li W, Lu L, Liu B, Qin S (2020). Effects of phycocyanin on pulmonary and gut microbiota in a radiation-induced pulmonary fibrosis model. Biomed Pharmacother.

[52] Ramos AF, Terry SA, Holman DB, Breves G, Pereira LG, Silva AG, Chaves AV (2018). Tucumã oil shifted ruminal fermentation, reducing methane production and altering the microbiome but decreased substrate digestibility within a RUSITEC fed a mixed hay–concentrate diet. Front Microbiol.

[53] Li Z, Liang H, Hu Y, Lu L, Zheng C, Fan Y (2023). Gut bacterial profiles in Parkinson’s disease: a systematic review. CNS Neurosci Ther.

