# Supplementary material for: Network analysis of gut microbial communities reveal key genera for a multiple sclerosis cohort with Mycobacterium avium subspecies paratuberculosis infection
Source: Gut Pathog. 2024 Jul 10;16:37. doi: 10.1186/s13099-024-00627-7 (PMC11238521; doi:10.1186/s13099-024-00627-7)
Supplement: Supplementary file 1 — Supplementary Material 1. Details on the network-wide statistics used including supplementary figures [file 13099_2024_627_MOESM1_ESM.docx]

**Supplementary Materials**

**Network inference of gut microbial communities in a multiple sclerosis cohort with Mycobacterium avium subspecies paratuberculosis infection**

Hajra Ashraf^1,4^, Plamena Dikarlo^2^, Aurora Masia^3^, Ignazio R. Zarbo^3^, Paolo Solla^3^, Umer Zeeshan Ijaz^4,5,6,*^, Leonardo A. Sechi^1,7,*^

^1^Department of Biomedical Sciences; University of Sassari, Sassari, Italy

^2^BIOMES NGS GmbH, Schwartzkopffstraße 1, 15745, Halle 21, Wildau, Germany

^3^Department of Medicine and Pharmacy, Neurology, University of Sassari, Italy

^4^Water & Environment Research Group, University of Glasgow, Mazumdar-Shaw Advanced Research Centre, Glasgow, United Kingdom

^5^National University of Ireland, Galway, University Road, Galway, Ireland

^6^Department of Molecular and Clinical Cancer Medicine, University of Liverpool, Liverpool, United Kingdom

^7^ Complex Structure of Microbiology and Virology; AOU Sassari, Sassari, Italy

* **Joint corresponding authors** (both authors jointly directed this work)

**Contact:**

Hajra Ashraf, [h.ashraf@studenti.uniss.it](mailto:h.ashraf@studenti.uniss.it)

Plamena Dikarlo, [plamena.dikarlo@biomes.world](mailto:plamena.dikarlo@biomes.world)

Aurora Masia, [masia.aurora@tiscali.it](mailto:masia.aurora@tiscali.it)

Ignazio R. Zarbo, [irzarbo@uniss.it](mailto:irzarbo@uniss.it)

Paolo Solla, [psolla@uniss.it](mailto:psolla@uniss.it)

Umer Zeeshan Ijaz*, [Umer.Ijaz@glasgow.ac.uk](mailto:Umer.Ijaz@glasgow.ac.uk) (<http://userweb.eng.gla.ac.uk/umer.ijaz>)

Leonardo A. Sechi*, [sechila@uniss.it](mailto:sechila@uniss.it)

Having obtained the network topologies, we have calculated several network wide statistics using numerous R packages including igraph [1], influential [2], and centiserve [3]. These are summarised in Supplementary Table S1.

**Supplementary Table S1**: Key centrality measures employed to ascertain influential nodes recovered in this study.

| Measure | Description | R’s Package |
| --- | --- | --- |
| Degree centrality | The degree centrality of a node is simply its degree - the number of edges it has. The higher the degree, the more central the node is. | igraph |
| Closeness centrality | How close a given node is to any other node, and defined as the inverse of the average of the shortest path between a node and all other nodes | igraph |
| Betweenness centrality | The number of shortest paths passing through a node. | igraph |
| Eigenvector centrality | The importance of a node is recursively related to the importance of the nodes pointing to it. A higher value implies that a node’s neighbours are more prestigious than the neighbours of other nodes | igraph |
| Subgraph centrality | It sums up all closed walks weighting them by the inverse factorial of its length | igraph |
| Coreness | The k-core of a graph is a maximal subgraph in which each vertex has at least degree k. The coreness of a vertex is k if it belongs to the k-core but not to the (k+1)-core | igraph |
| Kleinberg’s authority centrality score | The authority scores of vertices are defined as principal eigenvector of t(A)*A, where A is the adjacency matrix of the network | igraph |
| Eccentricity | Shortest path distance from the farthest other node in the graph | igraph |
| Neighborhood Connectivity | Average number of edges connected to immediate neighbors | influential |
| H-index | Semi-local centrality measure inspired from its application in assessing the impact of researchers | influential |
| Local H-index | An improved version of the H-index centrality that leverages the H-index to the second order neighbors of a node | influential |
| Spreading Score | Spreading score reflects the spreading potential of each node within a network, and is calculated as  ${Spreading}_{{score}_{i}}=({NC}_{i}^{'}+{CR}_{i}^{'})({BC}_{i}^{'}+{CI}_{i}^{'})$ where ${NC}_{i}^{'}$, ${CR}_{i}^{'}$, ${BC}_{i}^{'}$, ${CI}_{i}^{'}$ are range normalized neighborhood connectivity, cluster rank, betweenness centrality, and collective influence of node *i*. A node with high spreading potential will disproportionally disrupt more secondary nodes than those that have a low spreading potential. | influential |
| Collective Influence | A global centrality measure that calculates the product of the reduced degree (degree – 1) of a node and the total reduced degree of all nodes at a distance d from the node. | influential |
| Cluster Rank | ClusterRank is a local centrality measure that makes a connection between local and semi-local characteristics of a node and at the same time removes the negative effects of local clustering. | influential |
| Integrated View of Influence (IVI) | A method for the identification of network most influential nodes  in a way that captures all network topological dimensions, and is calculated as  ${IVI}_{i}=({Hubness}_{{score}_{i}})({Spreading}_{{score}_{i}})$ | influential |
| Hubness Score | Reflects the power of each node in its surrounding environment, and is calculated as ${Hubness}_{{score}_{i}}={DC}_{i}^{'}+{LH}_{{index}_{i}}^{'}$, where ${DC}_{i}^{'}$ and ${LH}_{{index}_{i}}^{'}$are ranged normalized degree centrality and local H index of node *i*, respectively. | influential |
| Topological coefficient | The extent to which a node shares neighbours with other nodes. | centiserve |
| Diffusion degree | The cumulative contribution score of the node itself and its neighbors. | centiserve |
| Density of maximum neighborhood component (DMNC) | It explores and identifies hubs/essential nodes in a network based on (23) | centiserve |
| Geodesic K-path centrality | Counts neighbours as those that are on a geodesic path less than "k" away | centiserve |
| Lin centrality | It considers closeness not as the inverse of a sum of distances, but rather than the inverse of the average distances | centiserve |
| Lobby Index | The l-index or lobby index of a node x is the largest integer k such that x has at least k neighbors with a degree of at least k | centiserve |
| Markov centrality score | The Markov centrality score uses the concept of a random walk through the graph to calculate the centrality of each vertex | centiserve |
| Latora closeness centrality | This variant (sum of inversed distances  to all other nodes instead of the inversed of the sum of distances to all other nodes) applicable to both connected and unconnected graphs | centiserve |
| Entropy centrality | Measures centrality of nodes depending on their contribution to the entropy of the graph | centiserve |
| Laplacian centrality | It is defined as the drop in the Laplacian energy (i.e. sum of squares of the eigenvalues in the Laplacian matrix) of the  graph when the vertex is removed. | centiserve |
| Leverage centrality | Leverage centrality considers the degree of a node relative to its neighbors and operates under the principle that a node in a network is central if its immediate neighbors rely on that node for information. | centiserve |
| Maximum neighborhood component (MNC) | The neighborhood of a node v, nodes adjacent to v, induce a subnetwork N(v).The score of node v, MNC(v), is defined to be the size of the maximum connected component of N(v). The neighborhood N(v) is the set of nodes adjacent to v and does not contain node v. | centiserve |
| Residual closeness centrality | Calculates the closeness of a vertex using (24) | centiserve |


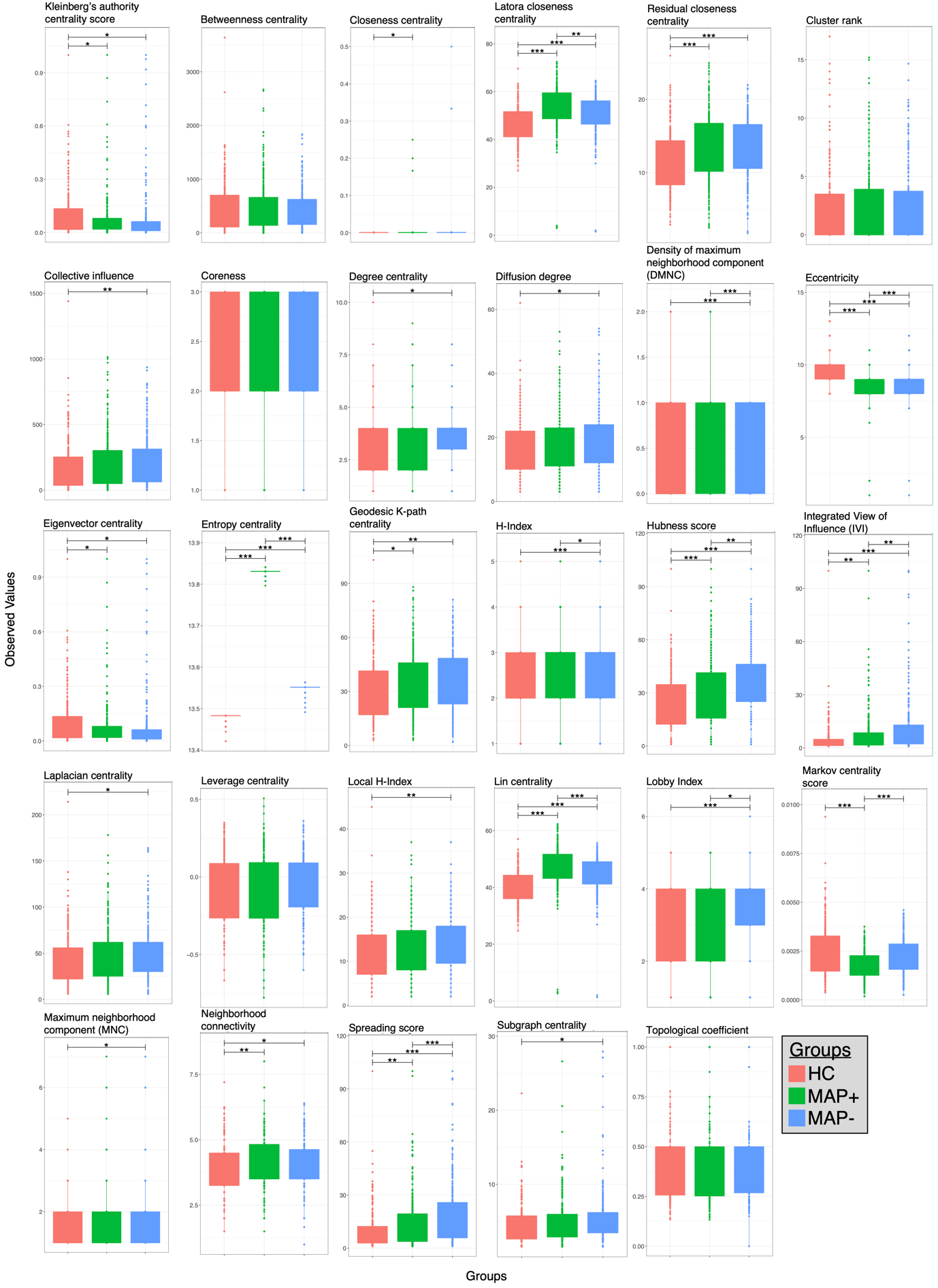


**Supplementary Figure S1**: Comparison of network-wide statistics for the networks obtained for HC, MAP+, and MAP- varieties. Lines for panels A and B connect two sample groups at statistically significant levels (ANOVA) indicated by asterisks as * (p < 0.05), **(p < 0.01) or ***(p < 0.001).

**Supplementary Figure S2**: 𝜷− coefficients returned from GLLVM procedure for covariates considered in this study, and the top 20 influential nodes returned for HC samples in Figure 1 using IVI metric. Those coefficients which are positively associated with the microbial abundance of a particular genera are represented in red color whilst those that are negatively associated are represented with blue color, respectively. Non-significant associations, if any, are represented with the black color. For categorical variables, one level acts as a reference and is annotated with REF. Genera also found influential for MAP+ and MAP- cohort are represented with red color, whilst those found in either of MAP- and MAP+ cohort are represented with green and blue colors, respectively.

**Supplementary Figure S3**: 𝜷− coefficients returned from GLLVM procedure for covariates considered in this study, and for the top 20 most influential nodes returned for MAP+ samples in Figure 2 using IVI metric. Those coefficients which are positively associated with the microbial abundance of a particular genera are represented in red color whilst those that are negatively associated are represented with blue color, respectively. Non-significant associations, if any, are represented with the black color. For categorical variables, one level acts as a reference and is annotated with REF. Genera also found influential for HC and MAP- cohort are represented with red color, whilst those found in either of MAP- and HC cohort are represented with green and blue colors, respectively.

**Supplementary Figure S4**: 𝜷− coefficients returned from GLLVM procedure for covariates considered in this study, and for the top 20 most influential nodes returned for MAP- samples in Figure 3 using IVI metric. Those coefficients which are positively associated with the microbial abundance of a particular genera are represented in red color whilst those that are negatively associated are represented with blue color, respectively. Non-significant associations, if any, are represented with the black color. For categorical variables, one level acts as a reference and is is annotated with REF. Genera also found influential for HC and MAP+ cohort are represented with red color, whilst those found in either of HC and MAP+ cohort are represented with blue color.

**References:**

1. Csardi, G., & Nepusz, T. (2006). The igraph software package for complex network research. InterJournal, complex systems, 1695(5), 1-9.
2. Salavaty, A., Ramialison, M., & Currie, P. D. (2020). Integrated value of influence: an integrative method for the identification of the most influential nodes within networks. Patterns, 1(5).
3. Jalili, M., Salehzadeh-Yazdi, A., Asgari, Y., Arab, S. S., Yaghmaie, M., Ghavamzadeh, A., & Alimoghaddam, K. (2015). CentiServer: a comprehensive resource, web-based application and R package for centrality analysis. PloS one, 10(11), e0143111.
